# Inter-Channel Error Calibration Method for Real-Time DBF-SAR System Based on FPGA

**DOI:** 10.3390/s25247561

**Published:** 2025-12-12

**Authors:** Yao Meng, Jinsong Qiu, Pei Wang, Yang Liu, Zhen Yang, Yihai Wei, Xuerui Cheng, Yihang Feng

**Affiliations:** 1 Department of Space Microwave Remote Sensing System, Aerospace Information Research Institute, Chinese Academy of Sciences, Beijing 100190, China; mengyao23@mails.ucas.ac.cn (Y.M.); qiujinsong@aircas.ac.cn (J.Q.); liuyang004301@aircas.ac.cn (Y.L.); 13661092882@139.com (Z.Y.); weiyihai22@mails.ucas.ac.cn (Y.W.); chengxuerui23@mails.ucas.ac.cn (X.C.); fengyihang23@mails.ucas.ac.cn (Y.F.); 2 School of Electronic, Electrical and Communication Engineering, University of Chinese Academy of Sciences, Beijing 100049, China

**Keywords:** DBF-SAR, channel error, pulse compression, FPGA

## Abstract

Elevation Digital Beamforming (DBF) technology is key to achieving high-resolution wide-swath (HRWS) imaging in spaceborne Synthetic Aperture Radar (SAR) systems. However, multi-channel DBF-SAR systems face a prominent conflict between the need for real-time channel error calibration and the constraints of limited on-board hardware resources. To address this bottleneck, this paper proposes a real-time channel error calibration method based on Fast Fourier Transform (FFT) pulse compression and introduces a “calibration-operation” dual-mode control with a parameter-persistence architecture. This scheme decouples high-complexity computations by confining them to the system initialization phase, enabling on-board, real-time, closed-loop compensation for multi-channel signals with low resource overhead. Test results from a high-performance Field-Programmable Gate Array (FPGA) platform demonstrate that the system achieves high-precision compensation for inter-channel amplitude, phase, and time-delay errors. In the 4-channel system validation, the DBF synthesized signal-to-noise ratio (SNR) improved by 5.93 dB, reaching a final SNR of 44.26 dB. This performance approaches the theoretical ideal gain and significantly enhances the coherent integration gain of multi-channel signals. This research fully validates the feasibility of on-board, real-time calibration with low resource consumption, providing key technical support for the engineering robustness and efficient data processing of new-generation SAR systems.

## 1. Introduction

With its unique advantages—including all-weather operation, high-resolution imaging, and strong penetration capabilities—Synthetic Aperture Radar (SAR) has become a cornerstone technology in numerous domains such as military reconnaissance, land and ocean monitoring, disaster management, and geographic mapping [1,2,3,4,5,6]. After decades of evolution, the performance of conventional SAR systems is approaching a bottleneck, yet the demand in practical applications for wider swath coverage and finer target resolution continues to grow. Consequently, the system design and signal processing techniques for new-generation High-Resolution Wide-Swath (HRWS) spaceborne SAR have become a primary research focus in the field [7,8,9,10,11,12,13,14,15,16,17,18,19,20]. Multi-channel SAR systems in elevation, by incorporating Digital Beamforming (DBF) technology [21,22,23,24,25], can generate receive beams with high gain and low sidelobes and scan the observed scene in real time. This capability significantly enhances the signal-to-noise ratio (SNR) for wide-swath imaging. However, the enhanced functionalities and superior performance of DBF-SAR [26,27,28] over conventional systems are underpinned by more complex signal processing algorithms and greater real-time data processing capabilities, which, in turn, present formidable challenges for hardware system design.

The deployment of multiple receive channels in the elevation direction results in a manifold increase in the volume of channel data acquired by the DBF-SAR system. Consequently, its data throughput is orders of magnitude greater than that of a conventional SAR; simultaneously, the DBF system is required to execute more complex functional models in real time. Considering that hardware resources on spaceborne platforms are typically subject to strict constraints, a prominent conflict emerges between the demands of high-performance data processing and the limited supply of on-board resources. Moreover, under the combined influence of factors such as complex electromagnetic environments, platform motion disturbances, and inter-channel hardware inconsistencies, the DBF-SAR system is highly susceptible to issues like phase errors and channel mismatch. These problems can subsequently lead to a degradation of imaging resolution and deviations in target localization.

Channel error has consistently been a core challenge in the radar domain. In early single-channel systems, these errors could be calibrated via offline, non-real-time processing after the data was downlinked to the ground and prior to image formation. For multi-channel systems, however, and particularly for DBF-SAR systems, resolving this issue presents a twofold dilemma. If the beamforming weights are stored on board the satellite, it consumes substantial hardware resources and restricts performance to a set of pre-stored modes. Conversely, if the multi-channel weight data is downlinked to the ground for processing, the operation is constrained by the limited satellite-to-ground transmission bandwidth, which impedes the implementation of real-time, closed-loop control. Consequently, the real-time synthesis of multi-channel signals must be performed on board. Against this backdrop, if channel errors cannot be calibrated in real-time at the hardware level, the error-laden signals will be combined, leading to a severe degradation in the quality of the synthesized data—a problem that is difficult to remedy through subsequent post-processing.

The conventional method for extracting inter-channel errors involves calculating the correlation of calibration reference data from different channels to further extract inter-channel difference information. On this basis, in the range-Doppler domain, the separation of signal subspace and noise subspace is achieved by performing eigenvalue decomposition on the covariance matrix; using the orthogonality of the two subspaces, high-precision estimation of inter-channel errors can be realized. However, existing subspace-based methods (e.g., Multiple Signal Classification (MUSIC) [29] and Estimation of Signal Parameters via Rotational Invariance Techniques (ESPRITs) [30]), although theoretically possessing super-resolution capability, rely heavily on covariance matrix calculation and iterative eigenvalue decomposition. These operations exhibit cubic computational complexity $O(N^{3})$ and non-deterministic convergence time, resulting in incompatibility with the stringent deterministic latency and power consumption constraints of the spaceborne Field-Programmable Gate Array (FPGA) platform. Consequently, they are unable to meet the requirements of on-board real-time processing.

To address the prominent conflict in multi-channel systems—where the high demand on hardware resources for on-board real-time calibration clashes with the inability of existing methods to meet real-time requirements—this paper introduces a novel, pulse compression-based scheme for real-time inter-channel error calibration in DBF-SAR. This method achieves on-board, real-time, closed-loop calibration with extremely low resource overhead, significantly enhancing the quality of the synthesized multi-channel signal. This work provides critical technological support for the practical implementation of HRWS spaceborne DBF-SAR and facilitates low-power, real-time processing. The structure of this paper is as follows: First, we elaborate on the origins of inter-channel errors in DBF-SAR and the theoretical basis for signal compensation, proposing a systematic error calibration methodology. Second, we provide a detailed description of the hardware architecture and implementation of the multi-channel error calibration module on an FPGA platform. Finally, the effectiveness of the proposed method is validated through experimental tests that verify the performance metrics of the calibrated signal.

## 2. Error Calibration Method

### 2.1. Analysis of the Causes of Channel Errors

The causes of inter-channel errors in DBF-SAR systems are multifaceted, stemming from both hardware non-idealities and dynamic environmental factors. Beyond basic manufacturing variations and transmission path discrepancies, factors such as ionospheric Doppler effects, clock skew, analog-to-digital conversion (ADC) nonlinearities, and quantization noise significantly impact signal integrity. However, from the perspective of signal modeling and real-time calibration, these diverse physical mechanisms primarily manifest as deviations in the channel transfer function’s three core parameters.

Specifically, component aging (long-term) and operational temperature drifts (short-term) principally induce amplitude fluctuations and phase deviations in the analog front-end. Meanwhile, synchronization discrepancies during the initialization of sampling clocks across multiple ADCs, along with clock distribution skew, directly result in inter-channel sampling time delays. Although ionospheric perturbations introduce additional time-varying phase errors and ADC nonlinearities contribute to signal distortion, within the context of channel consistency calibration, these cumulative effects are effectively modeled as a three-dimensional error vector consisting of amplitude mismatch, phase deviation, and time delay. Therefore, accurate extraction and compensation of these three parameters are sufficient to mitigate the dominant effects of the aforementioned complex physical factors.

Although inter-channel errors are manifested as single-point numerical values, this type of systematic deviation directly compromises the coherent integration characteristics of the multi-channel signals. Under ideal conditions, the objective of multi-channel signal synthesis is to achieve coherent superposition, thereby obtaining the maximum array processing gain. For an ideal N-channel system, the coherent integration gain in the power domain should satisfy the criterion of $G=10\log_{10}N\;\mathrm{dB}$, (where *N* is the number of channels). Consequently, the synthesized SNR should be proportional to $SNR\_sys=SNR\_single+10\log_{10}N$. However, the presence of inter-channel errors causes the actual gain to fall significantly below the theoretical value and can even lead to coherent cancellation, degrading the SNR. To quantitatively analyze the actual impact of channel errors on the synthesized SNR, this study conducts a simulation using the system parameters presented in Table 1.

The ranges of the randomly introduced amplitude, phase, and sampling-grid time-delay errors are given in Table 2.

To quantitatively evaluate the impact of channel errors on system performance, the simulation experiment introduces randomly set amplitude, phase, and sampling delay error terms into each signal channel. Subsequently, the SNR is analyzed for each signal after pulse compression, and this is compared with the SNR obtained after first performing DBF synthesis and then applying pulse compression. The relevant results are presented in Table 3:

As is evident from Table 3, the SNR of the DBF-synthesized signal under these conditions shows an improvement of only 8.4759 dB relative to a single channel. This represents a significant deviation from the ideal result and validates the adverse impact of channel errors. This finding underscores that if channel errors are not effectively compensated, the performance advantages of multi-channel synthesis will remain unrealized, severely compromising the engineering robustness of the system design.

### 2.2. The Principle of Error Calibration Algorithm

Traditional internal calibration injects a reference signal into the receive chain via a coupler and estimates the amplitude, phase, and delay errors of each channel along a dedicated calibration path. However, this approach has three fundamental limitations. First, to avoid ADC saturation and intermodulation distortion, the injection power must be constrained; together with losses from coupling/splitting/switching, this yields a low equivalent SNR in the calibration branch. For a given noise power spectral density and receive bandwidth, the back-end SNR of the calibration echo is therefore typically lower than that of the operational echoes. Second, because the calibration branch is not common-path with the imaging signal, additional amplitude and phase biases are introduced that do not faithfully represent the imaging chain. Third, short-term drifts caused by on-board temperature variations, load changes, and switch states cannot be reliably tracked by the intermittent nature of internal calibration measurements. As a result, accuracy is limited, representativeness for the imaging link is insufficient, and the equivalent SNR of the imaging data is not substantially improved. The problem is exacerbated under weak-echo/strong-noise conditions, where the upper bound on injection power further degrades parameter-extraction accuracy.

In view of the complex system errors and instantaneous channel inconsistencies existing in the DBF-SAR system, this paper proposes a real-time pulse compression calibration algorithm based on Fast Fourier Transform (FFT) implementation. The algorithm makes full use of the large time-bandwidth product characteristic of linear frequency modulation (LFM) signals, and obtains significant SNR gain through matched filtering processing, thereby achieving high-precision error extraction in a low SNR environment.

#### 2.2.1. Pulse Compression and SNR Analysis

The core of this algorithm lies in focusing signal energy using frequency-domain pulse compression, thereby achieving effective separation of signals and noise. According to radar signal processing theory, after the LFM signal undergoes matched filtering, there is the following gain relationship between its output SNR $SNR_{out}$ and input SNR $SNR_{in}$:

(1) $$G_{pc}=\frac{SNR_{out}}{SNR_{in}}\approx 10\log_{10}\left(T_{p}\cdot B\right)$$

where $T_{p}$ is the signal pulse width and *B* is the signal bandwidth. In the simulation parameter setting of this system ($T_{p}$ = 10 µs, B=40MHz), the time-bandwidth product $T_{p}B=400$. This means that the system can provide a signal-to-noise ratio gain of approximately 26 dB. Therefore, even in the original 10 dB signal-to-noise ratio environment set in the simulation, the peak signal-to-noise ratio after pulse compression can be increased to more than 36 dB. This high signal-to-noise ratio characteristic theoretically ensures the extremely low variance of the extraction of amplitude, phase and delay parameters, and effectively overcomes the defect that traditional methods are sensitive to noise.

#### 2.2.2. Algorithm Processing Flow and Mathematical Model

**Figure 1:**
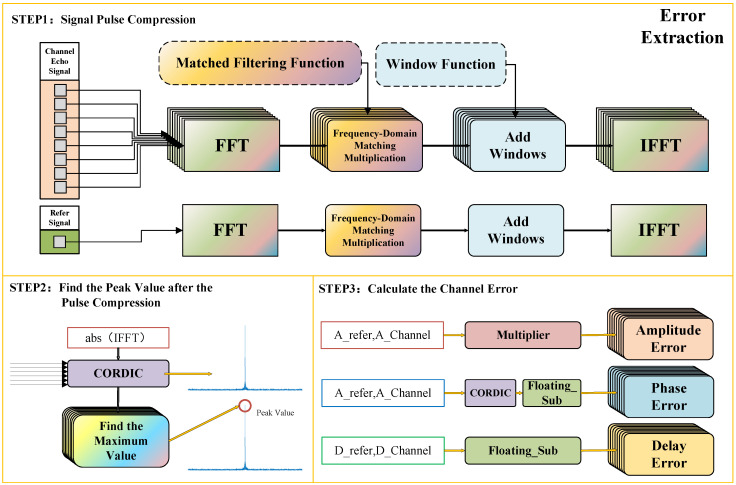
The process of the channel error calibration algorithm.

The specific flow of the algorithm is shown in Figure 1. To further suppress sidelobe interference and prevent strong sidelobes from causing peak detection ambiguity in a multi-channel environment, this algorithm introduces Hamming Window weighting processing in the frequency-domain matching process.

Let the echo signal of the *k*-th channel be $r_{k}\left[n\right]$, the reference signal be $s_{\mathrm{ref}}\left[n\right]$, and the frequency-domain response of the matched filter be H(ω) (determined by the transmitted waveform). First, perform FFT transformation on the echo signal of each channel to the frequency domain:

(2) $$S_{k}\left(\omega \right)=\mathrm{FFT}\left\{r_{k}\left[n\right]\right\}$$

While performing matched filtering in the frequency domain, multiply by the Hamming window function W(ω) to suppress time-domain sidelobes. The modified frequency-domain processing model is as follows:

(3) $$Y_{k}\left(\omega \right)=S_{k}\left(\omega \right)\cdot H\left(\omega \right)\cdot W\left(\omega \right)$$

where the time-domain window function corresponding to W(ω) is w(n)=0.54−0.46cos(2πn/(N−1)), which can reduce the peak sidelobe level, thereby eliminating the interference of sidelobes on the main peak detection.

Subsequently, the signal is restored to the time domain through Inverse Fast Fourier Transform (IFFT), and the complex signal sequence after pulse compression is obtained:

(4) $$y_{k}\left[n\right]=\mathrm{IFFT}\left\{Y_{k}\left(\omega \right)\right\}$$

Similarly, perform the same windowed pulse compression processing on the ideal reference signal:

(5) $$p\left[n\right]=\mathrm{IFFT}\left\{\mathrm{FFT}\{s_{\mathrm{ref}}\left[n\right]\}\cdot H\left(\omega \right)\cdot W\left(\omega \right)\right\}$$

Search for the maximum modulus value and its position in the sequence after pulse compression, and extract phase information:

(6) $$a_{k}\;=\;\max_{n}\;|y_{k}\left[n\right]|$$

(7) $$n_{k}\;=\;\mathrm{sample}\;\left(\max_{n}\;|y_{k}\left[n\right]|\right)$$

(8) $$\phi_{k}\;=\;∠\;\left(\max_{n}\;|y_{k}\left[n\right]|\right)$$

The reference sequence is the same:

(9) $$a_{\mathrm{ref}}\;=\;\max_{n}\;|p\left[n\right]|$$

(10) $$n_{\mathrm{ref}}\;=\;\mathrm{sample}\;\left(\max_{n}\;|p\left[n\right]|\right)$$

(11) $$\phi_{\mathrm{ref}}\;=\;∠\;\left(\max_{n}\;|p\left[n\right]|\right)$$

Using the extracted parameters, calculate the error of the *k*-th channel relative to the reference signal. Amplitude error factor:

(12) $${\hat{A}}_{k}\;=\;\frac{a_{\mathrm{ref}}}{a_{k}}$$

The phase error is the following:

(13) $${\hat{\phi }}_{k}\;=\;\phi_{\mathrm{ref}}\;-\;\phi_{k}$$

With the time delay error represented as follows:

(14) $${\hat{\tau }}_{k}\;=\;n_{\mathrm{ref}}\;-\;n_{k}$$

In the actual spaceborne environment, in addition to hardware errors, non-ideal factors such as Doppler frequency shift caused by platform motion will also affect the echo signal. However, considering that the aperture scale of the spaceborne DBF antenna array is much smaller than the target slant range, the Doppler effect manifests as a highly consistent overall time shift and frequency shift (common-mode errors) among each receiving channel. Since the calibration algorithm proposed in this paper is based on the differential extraction of relative parameters between channels (i.e., calculating the difference between channel *k* and the reference channel), the aforementioned common-mode errors will cancel each other out during the calculation process. Therefore, the Doppler frequency shift does not affect the accuracy of the relative amplitude-phase consistency between channels, and the corrected signal can still ensure the efficiency of beamforming.

By introducing the pulse compression gain mechanism and frequency-domain windowing suppression method, the proposed algorithm significantly improves the anti-noise performance and anti-interference capability while ensuring low resource consumption. It not only solves the real-time problem of traditional methods but also ensures the calibration accuracy in complex channel environments.

### 2.3. Design Scheme of DBF-SAR Error Calibration System

**Figure 2:**
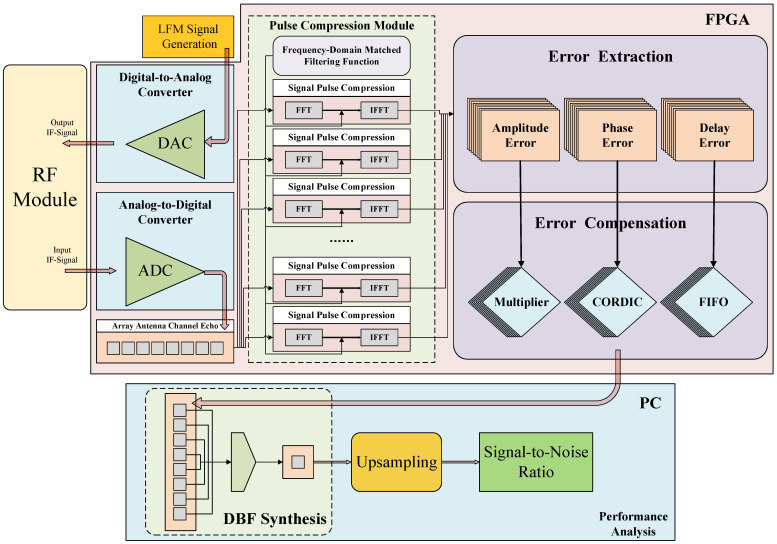
DBF-SAR channel error calibration system.

The architecture of the DBF-SAR channel error calibration system designed in this paper is illustrated in Figure 2. The system is composed of three main parts: a radio frequency (RF) module, an FPGA digital signal processing board, and a PC processor. The RF module provides power for the digital signal processing board. The FPGA functions as the core unit for real-time digital signal processing, integrating several key modules for digital-to-analog conversion(DAC), ADC, signal acquisition, pulse compression, error extraction, and error compensation. This allows for the efficient, low-latency, real-time processing of multi-channel echo signals. In contrast, the PC processor is used for non-real-time, high-level data processing tasks—specifically DBF synthesis and upsampling—which provide the data foundation for final image formation and performance analysis.

The system’s high-performance DAC and ADC can directly achieve the output and sampling of intermediate frequency signals. This design effectively simplifies the hardware structure, adapting to the integration requirements of on-board systems. The error extraction and error compensation modules are the core carriers of the channel error calibration scheme proposed in this paper, and their performance directly determines the system’s overall calibration accuracy. Finally, the compensated signals from multiple channels are transmitted to the PC computing unit to complete the system performance verification.

**Figure 3:**
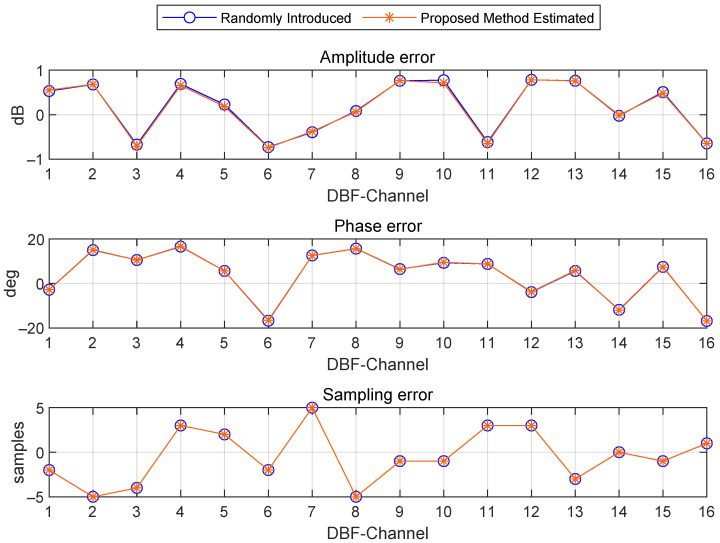
Error estimation results.

The real-time error extraction method adopted in this research is based on a direct echo signal processing mechanism. Its technical principle is to achieve the joint estimation of multi-dimensional error parameters by resolving the time–frequency characteristics derived from pulse compression. Specifically, the method leverages the time–frequency coupling property of the LFM signal, performing pulse compression via matched filtering. This process makes it possible to decouple key error parameters—namely the amplitude mismatch factor $\Delta \alpha_{i}$, the phase deviation term $\Delta \varphi_{i}$, and the relative time delay $\Delta \tau_{i}$—within the joint time–frequency domain. Figure 3 shows the comparison result between the error extracted by this method and the channel error initially set by the system.

**Figure 4:**
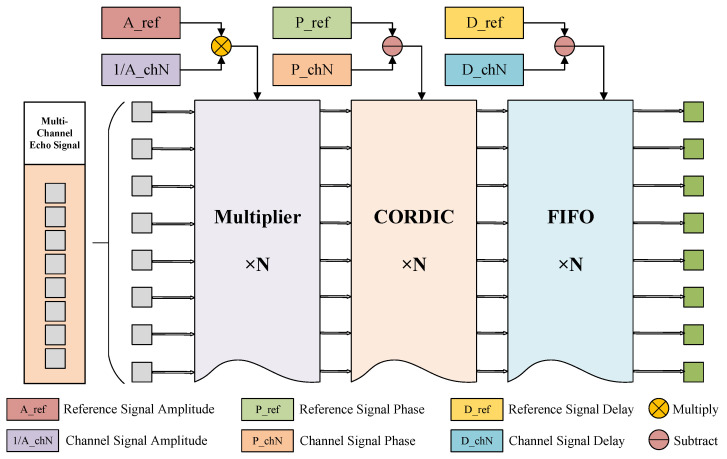
Flowchart of channel error compensation.

After extracting the inter-channel amplitude, phase, and time delay error information, compensation must be applied to each channel. The real-time compensation method adopted in this paper is illustrated in Figure 4.

Employs targeted implementation approaches for different types of error: amplitude error is calibrated through multiplicative compensation by adding a multiplier in each path, in the FPGA design, the calibration process uses a 32-bit width (with intermediate accumulation extended to 64 bits); phase error is compensated using a CORDIC module to perform digital phase shifting; and for sampling time delay error, the compensation of inter-channel time delay errors is achieved by combining the dynamic adjustment of sampling timing alignment (via a programmable FIFO). All of the above methods can effectively compensate for the system’s inter-channel errors with minimal additional resource overhead, making the scheme highly feasible and low in complexity under resource constraints.

It is worth noting that in physical hardware, time delay and phase error are correlated, since a time delay τ induces a carrier phase shift $\Delta \phi =2\pi f_{c}\tau$. The proposed method addresses this coupling issue by extracting the total phase error observed at the pulse compression peak. The phase ${\hat{\phi }}_{k}$ extracted in Equation (13) naturally includes the inherent hardware phase deviation and the phase shift caused by time delay. Therefore, the application of phase compensation (via CORDIC) eliminates the composite phase error. Meanwhile, digital time delay compensation (via FIFO) aligns the signal envelope. Since digital integer shift does not introduce additional phase rotation, these two compensation steps can be performed independently, thereby achieving complete calibration of the correlated errors.

After completing the channel error compensation, to verify the effect of the compensation, the SNR of the single-channel pulse compression result was again compared with the SNR of the pulse-compressed signal after DBF synthesis. The relevant results are shown in Table 4.

As shown in Table 4, after compensating for the inter-channel amplitude, phase, and sampling delay errors, the SNR of the DBF-synthesized signal improved by 11.6474 dB relative to a single channel. Theoretically, for an *N*-channel signal synthesis, the SNR should increase by $10\log_{10}N\;\mathrm{dB}$; for this system, (where *N* = 16, the ideal theoretical gain is 12.0412 dB). The low deviation between the measured improvement and this ideal gain fully validates the high precision and engineering effectiveness of the proposed pulse compression-based error extraction and real-time inverse compensation scheme.

## 3. Experiments and Results

### 3.1. Hardware Implementation and Real-Time Optimization

In a DBF system, inter-channel amplitude and phase inconsistency are critical factor that affect system performance. To achieve high-precision compensation for the received signals, this design employs a three-stage processing pipeline comprising Pulse Compression, Error Extraction, and Error Compensation. However, because the pulse compression process involves complex computations such as the FFT/IFFT, it introduces a significant, fixed pipeline latency. If this high-latency calibration process were to be repeatedly executed on a frame-by-frame basis during normal system operation, it would severely limit the system’s computational efficiency and data throughput rate.

To resolve this real-time bottleneck and adapt to dynamic variations in the spaceborne environment, this paper proposes a hardware architecture optimization scheme based on a “calibration-operation” dual-mode control and parameter persistence. This scheme decouples the high-complexity, high-latency error extraction computation by confining it to periodic calibration operations (e.g., performed during system initialization or intervals between data takes). Given that environmental factors such as thermal drift are typically slow-varying processes, this approach achieves “intermittent computation with persistent coefficient storage for compensation.” This strategy not only effectively tracks time-varying drifts in channel parameters but also significantly reduces processing latency and resource consumption during the system’s stable operational mode (i.e., the data acquisition and synthesis phase).

**Figure 5:**
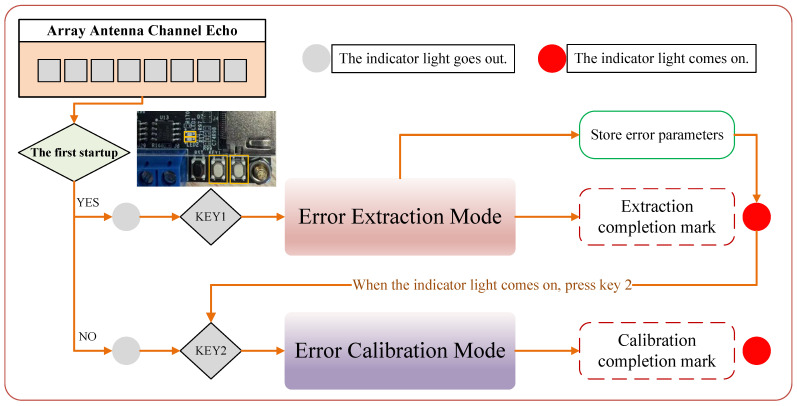
Shows the FPGA flowchart based on the “calibration-operation” dual-mode control.

The FPGA hardware architecture employing a “calibration-operation” dual-mode control with parameter persistence is illustrated in Figure 5. When Key 1 is pressed, the channel echo enters the error-extraction mode, where amplitude, phase, and timing (delay) errors are estimated. Upon completion, Indicator LED 1 turns on, and the extracted error parameters are latched into registers for persistent storage and subsequent compensation. Pressing Key 2 switches the input channel echo to the error-compensation mode: the signal is then routed only through the calibration module, bypassing pulse compression and other high-latency processing, which substantially reduces per-calibration latency and thereby ensures real-time operation.

### 3.2. Simulation Experiment Results

**Figure 6:**
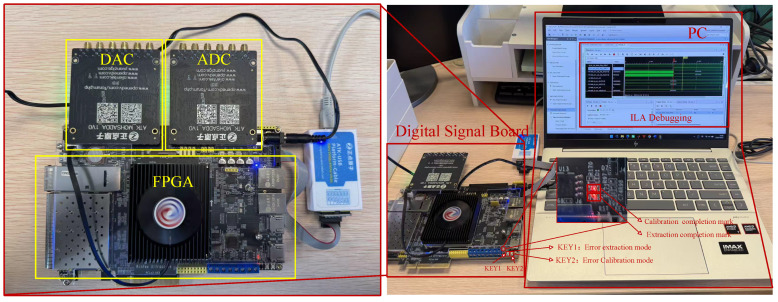
Hardware platform of the ground test system.

**Figure 7:**
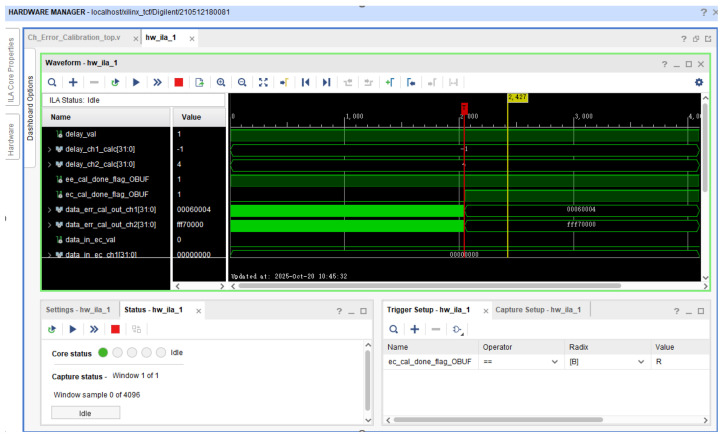
ILA simulation debugging.

To verify the performance of the DBF-SAR real-time inter-channel error calibration system, the research team constructed an equivalent 4-channel test platform in a ground environment to conduct echo signal tests. This paper selects the high-performance Kintex Ultrascale series FPGA chip (KU040) manufactured by Xilinx (San Jose, CA, USA) as the hardware platform. This chip is equipped with abundant logic resources, high-speed data processing capabilities, and flexible reconfigurable characteristics, which can fully meet the requirements of the DBF-SAR system for real-time performance and complexity. As shown in Figure 6, the ground test system consists of a computer and an FPGA digital signal processing board. The Integrated Logic Analyzer (ILA) online debugging process is shown in Figure 7.

To fully evaluate the calibration performance, errors in all three dimensions—amplitude, phase, and time delay—were simultaneously injected into the channel signals. The scope and characteristics of these errors are consistent with the simulation parameters previously defined in Table 2. During the testing process, the computer sends control instructions to the digital signal board. Upon receiving these instructions, the high-performance FPGA first generates a reference LFM signal. This signal is output via the DAC and looped back to the ADC input, and within this transmission path, channel errors are either injected externally or emulated internally by the FPGA to simulate the error-laden echo signals received by a DBF-SAR. Subsequently, the FPGA digital signal board completes the real-time channel error extraction and compensation operations. The calibrated data from the four channels are then transmitted to the PC processor, where subsequent non-real-time processing tasks, such as DBF synthesis and performance analysis, are executed.

**Figure 8:**
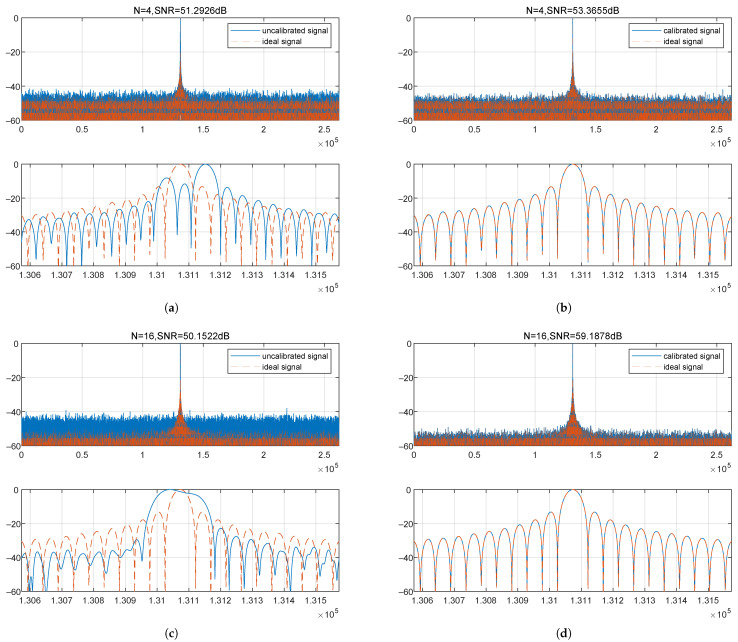
Impact of channel error on the point target response and signal-to-noise ratio (SNR). (**a**) *N* = 4, DBF with channel errors. (**b**) *N* = 4, DBF after channel error calibration. (**c**) *N* = 16, DBF with channel errors. (**d**) *N* = 16, DBF after channel error calibration.

Figure 8 intuitively displays a comparison of the multi-channel synthesized pulse compression results after real-time calibration with those from signals containing channel errors.

In this simulation case, the single-channel post-pulse-compression SNR is approximately 47.35 dB; a 4-channel configuration should provide a 6.02 dB gain, and a 16-channel configuration should provide a 12.04 dB gain. Figure 8a shows that in the presence of channel errors, an SNR improvement of only 3.94 dB is provided, and Figure 8c shows an SNR improvement of only 2.8 dB. After error calibration, the system successfully achieves its designed performance metrics (Figure 8b,d). By quantitatively calculating the key performance metric of SNR before and after calibration, this paper further validates the high-precision compensation capability and excellent performance of the DBF-SAR real-time channel calibration system.

### 3.3. Engineering Constraints and Power Analysis for Spaceborne Platforms

Considering the stringent Size, Weight, and Power (SWaP) constraints of the spaceborne platform, this design conducts a detailed evaluation of the resource utilization and power consumption of the FPGA implementation.

(1) Resource Utilization: As shown in Table 5 and Table 6, on the Kintex Ultrascale FPGA, the occupancy rates of LUT (core logic) and DSP units of the error calibration system are only 5.7% and 3.48%, respectively. This extremely low resource utilization rate (approximately 10%) holds significant engineering significance: it not only avoids the congestion risk during large-scale multi-channel parallel processing but also reserves sufficient chip space for the subsequent integration of more complex imaging algorithms and other designs.
(2) Power Consumption Analysis: The power consumption analysis report based on Vivado Post-Implementation shows that at an operating frequency of 100 MHz for the system clock, the total on-chip power consumption of the real-time error calibration module is only 0.963 W, among which the dynamic power consumption caused by logic flipping and signal processing is 0.333 W, and the static leakage power consumption of the device is 0.63 W. This scheme significantly reduces energy consumption while ensuring accuracy. For the spaceborne environment that is extremely sensitive to thermal design, this low-power characteristic means that the pressure on the thermal management subsystem can be reduced, and valuable power budgets can be reserved for other high-throughput data processing tasks on the satellite, fully verifying the robustness and feasibility of the scheme in engineering applications.
(3) Real-time Constraints: Compared with high-complexity iterative algorithms, this design is based on a pipelined architecture, and the processing latency from data input to error parameter extraction is approximately 300 µs. This deterministic low-latency characteristic ensures that the system can adapt to the high Pulse Repetition Frequency (PRF) operating mode and meet the real-time constraints of HRWS imaging.

**Table 5:** The utilization of synthesis design resources.

Resource	Estimation	Available	Utilization %
LUT	36,793	331,680	11.09
LUTRAM	29,696	146,880	20.22
FF	10,461	663,360	1.58
BRAM	16	1080	1.48
IO	7	520	1.35
BFUG	1	624	0.16

**Table 6:** The utilization of implementation design resources.

Resource	Utilization	Available	Utilization %
LUT	18,902	331,680	5.70
LUTRAM	1659	146,880	1.13
FF	23,209	663,360	3.50
DSP	97	2760	3.48
IO	7	520	1.35
BFUG	7	624	1.12

## 4. Conclusions

This paper investigates the channel error problem in the engineering practice of DBF-SAR systems, which arises from multi-channel hardware non-idealities and environmental perturbations, and proposes a real-time calibration method for inter-channel amplitude, phase, and time delay based on frequency-domain pulse compression. By introducing a “calibration-operation” dual-mode control and parameter persistence architecture, the method effectively confines high-complexity computations to the initialization phase, achieving high-precision, real-time compensation of multi-channel signals with extremely low hardware resource overhead. This enables the resolution of mismatch issues introduced by the channel links prior to DBF synthesis. Experimental results demonstrate that after eliminating channel errors, the SNR of the DBF-synthesized signal is improved by 5.93 dB, which is a performance level that approaches the ideal theoretical value of 6.02 dB. This fully validates the significant enhancement effect of the proposed scheme on the final imaging quality of the system. The findings of this study successfully validate the feasibility of on-board, real-time channel calibration with low resource consumption, providing important technical support and a practical reference for the future engineering deployment of new-regime SAR systems and for the design of data links for high-resolution, wide-swath imaging. While the point target analysis confirms the theoretical validity and high precision of the proposed method, we acknowledge that strong ground clutter and dynamic scene variations in real spaceborne missions may introduce additional challenges. Future work will be dedicated to verifying this architecture using airborne multi-channel SAR data and investigating its performance in complex clutter environments to further enhance its engineering robustness.

## Figures and Tables

**Table 1:** Simulation system parameters.

Parameter	Value
Carrier Frequency	9.65 GHz
Orbit Height	567 km
Sample Rate	240 MHz
Signal Pulse Duration	10 µs
Signal Bandwidth	40 MHz
Antenna Installation Angle	$25^{\circ }$
Number of Channels	16
Signal-to-Noise Ratio	10 dB
Look Angle of the Antenna Normal Direction	23∼27°

**Table 2:** Simulation error parameters.

Error Type	Scope	Unit
Amplitude Error	−1∼1	dB
Phase Error	−20∼20	°
Time-Delay Error	−5∼5	Sampling Point

**Table 3:** Signal-to-noise ratio analysis of uncalibrated errors.

Object	SNR (dB)
Channel 1	38.1559
Channel 2	38.2921
…	…
Channel 16	38.1219
DBF Synthetic Result	46.7913
Average Improvement	8.4759

**Table 4:** Analysis of signal-to-noise ratio after error compensation.

Object	SNR (dB)
Channel 1	38.1559
Channel 2	38.2921
…	…
Channel 16	38.1219
DBF Synthetic Result	49.9628
Average Improvement	11.6474

## Data Availability

The original contributions presented in this study are included in the article. Further inquiries can be directed to the corresponding author.
